# Gestational age determination in neonates - transcerebellar ultrasound measurements help: a retrospective study of 671 neonates

**DOI:** 10.1007/s00247-025-06426-9

**Published:** 2025-11-19

**Authors:** Preeti S. Prasad, Harris L. Cohen, Minhee Jo, Mimily Harsono, Liu-Smith Feng, Chenhao Zhao, Massroor Pourcyrous

**Affiliations:** 1 Department of Radiology, Le Bonheur Children’s Hospital, 848 Adams Ave, Memphis, TN 38103 USA; 2 University of Tennesse Health Science Center, Memphis, TN USA; 3 The University of Texas Health Science Center at Houston, Houston, TX USA

**Keywords:** Cerebellum, Gestational age, Infant, Neonate, Newborn, Premature birth, Ultrasonography

## Abstract

**Background:**

Many children are born without prenatal determination of gestational age (GA). Postnatal determinations are limited. Fetal GA determination using transcerebellar diameter measurements is reliable for fetuses. We wanted to see if transcerebellar diameters obtained on neonatal head ultrasound exams could help GA determination in newborns and whether such measurements conformed to similar GA determinations in fetuses.

**Objective:**

Our goal was to determine if neonatal GA can be estimated by measuring transcerebellar diameter via a transmastoid approach using fetal charts as the gold standard. If true, one could develop a neonatal chart for GA determination by transcerebellar diameter.

**Materials and methods:**

Transmastoid views are a routine part of our neonatal intensive care unit neurosonograms. A retrospective analysis of transcerebellar diameters of neonates (1 day to 21 days old) born between 22 weeks and 40 weeks corrected GA was performed. Cases with congenital anomalies, intraventricular hemorrhage, or other neurosonographic abnormalities were excluded. Neonatal GA was determined by early antenatal crown rump lengths. We calculated transcerebellar mean and standard deviation for each prenatally determined GA week. GA was determined from fetal charts, both for subsets of neonates evaluated at less than or equal to (≤) 10 days of life and for those examined at ≤21 days of life. Statistical analysis using linear regression demonstrated no differences in GA determined by neonatal transcerebellar diameter compared to fetal charts (our gold standard).

**Results:**

We evaluated 1,260 neurosonograms. Of these, 589 cases were excluded. A total of 671 exams were of neonates ≤21 days old; 530 of those were examined at ≤10 days of life. There were no significant differences between GA determined by fetal charts and our neonatal transcerebellar diameters, whether from the ≤21-day (*P*=0.15) or the younger ≤10-day group (*P*=0.87).

**Conclusion:**

Neonatal GA estimation by transmastoid fontanelle measurements of cerebellar width appears as reliable as the accepted antenatal transcerebellar measurements of fetuses. Our proposed neonatal chart will hopefully aid reliable estimation of GA in neonates, improving patient care among neonates with unknown maternal last menstrual period.

**Graphical abstract:**

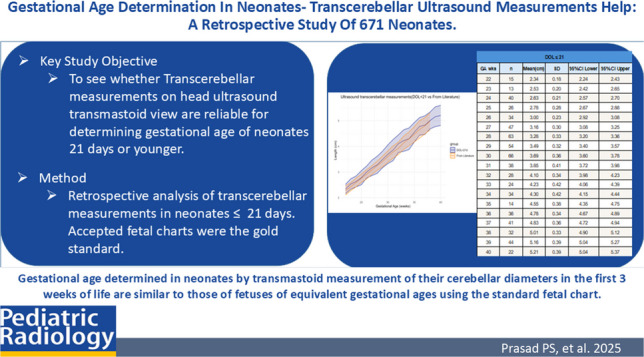

## Introduction

Knowledge of the true gestational age of neonates at the time of their delivery is important and a necessity for their care. Correct information regarding the gestational age of neonates in the intensive care unit affects their clinical diagnoses and treatment plans [1]. There are times when the gestational age of a newborn cannot be determined, either due to a history of irregular periods or the unknown last menstrual period of the mother or due to the nonexistence or nonavailability of early fetal imaging measurements. Based on the best documented estimated gestational age, clinicians use the Fenton growth chart and correlate gestational age with weight, length, and head circumference that were measured after birth. Having the best estimated gestational age helps determine whether a neonate’s growth is appropriate for their gestational age or suggests the possibility of symmetric or asymmetric intrauterine growth retardation. It can suggest a congenital, chromosomal, or infectious neonatal cause vs placental malfunction. Knowledge of gestational age helps assess neonatal risk for respiratory distress syndrome or for necrotizing enterocolitis. Gestational age is essential in determining the risk of intraventricular hemorrhage in the neonates.

In our study, the transcerebellar diameter was chosen because it is a proven helpful measurement for gestational age determination in fetuses. In addition, the cerebellum is one of the least intracranial organs affected by fetal growth retardation, as discussed in the article by Makhoul IR et al. [2]. There are accepted charts in the obstetric literature to determine the gestational age of a fetus by measuring the transcerebellar diameter on antenatal ultrasound [3–5]. No such data or charts have been created or are available for neonates. Currently, the New Ballard Score is the most widely accepted method to assess a neonate’s postnatal gestational age [6–8]. It is a clinical method based on physical and neuromuscular maturity and can be used up to 4 days after birth. However, the New Ballard Score has limitations, especially in early preterm neonates, where the gestational age could be overestimated [9].

The goal of this study is to examine whether a transcerebellar diameter obtained by transmastoid views in a neonate can be used for newborn gestational age determination.

## Materials and methods

This was a retrospective study approved by the institutional review board. The research plan was to obtain transcerebellar measurements via the transmastoid view among neonates with known gestational ages and compare them to accepted measurements for gestational age determination among fetuses of similar gestational age weeks. If found statistically similar, a chart could be developed depicting transcerebellar measurements for each studied gestational age week to be used for neonates of unknown gestational age.

The subjects were neonates between 22 weeks and 40 weeks of gestation. We performed a retrospective analysis of neonates evaluated by head ultrasound in our neonatal intensive care unit over a 5-year period between 2015 and 2020.

**Fig. 1:**
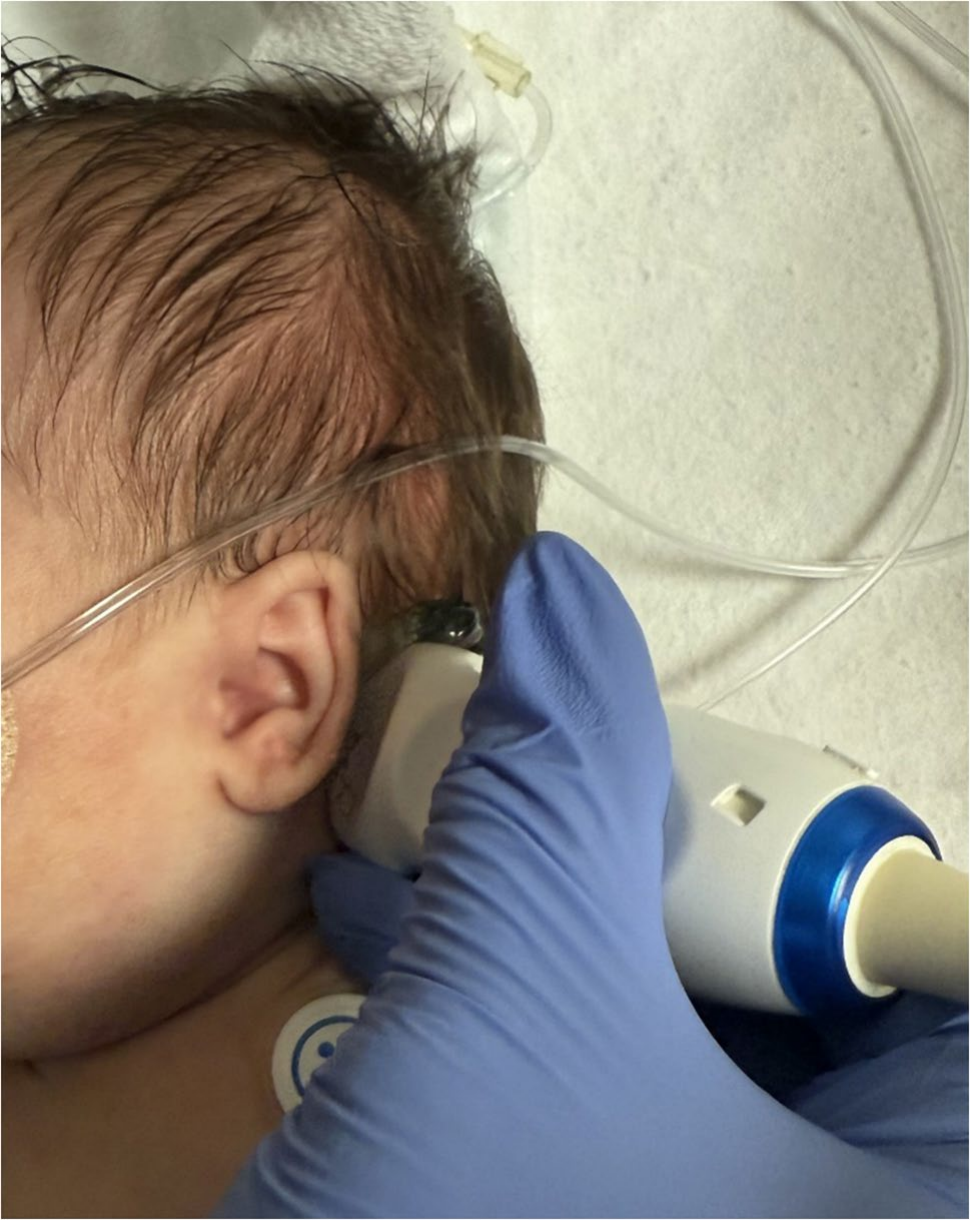
Transmastoid ultrasound being performed on a 10-day-old girl born at 34 weeks of gestation by placing a micro-convex array transducer on the mastoid fontanelle just behind the left ear. The transducer is swept and angulated to obtain a symmetrical image of the cerebellar hemispheres

**Fig. 2:**
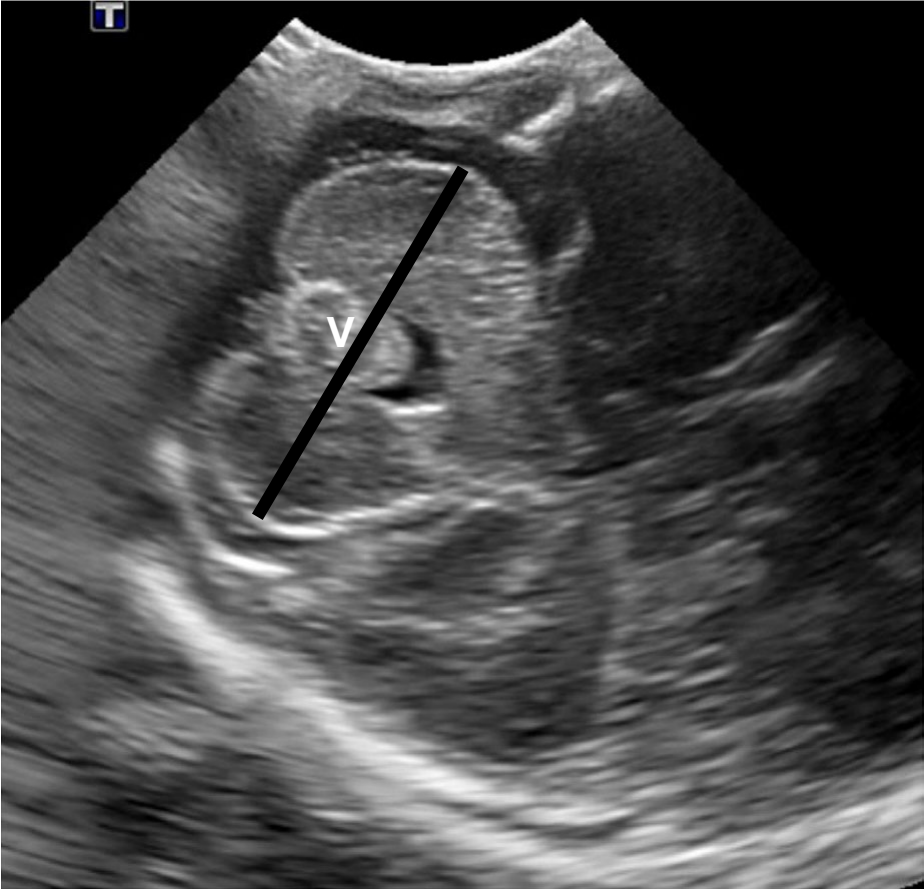
Transmastoid ultrasound image of a 7-day-old male born at 35 weeks of gestation showing symmetrical cerebellar hemispheres. The *black line* demonstrates the greatest width from the lateral aspect of one cerebellar hemisphere to the other side. “*V*” depicts the cerebellar vermis

Our neonatal neurosonography protocol classically used transfontanelle imaging via the anterior fontanelle. Beginning in 2015, we began to add transmastoid views to improve the diagnosis of the subtentorial area and its contents. Currently, transmastoid imaging is a part of our institution’s routine head ultrasound protocol. The exams were performed on a Toshiba Aplio i800 ultrasound machine with a PVT-712BT 11MC4 micro-convex Ultrasound Probe (4.2–10.2 MHz). The images are obtained either via the right mastoid or via the left mastoid approach, and never both. Transmastoid ultrasound is performed by placing a high-frequency curvilinear array transducer with a small footprint at the right or left mastoid fontanelle—just behind the ear (Fig. 1). The transducer is swept and angulated to obtain the best symmetrical axial image of the cerebellar hemispheres with a central cerebellar vermis. Using a single symmetric transmastoid image, a single transcerebellar measurement at its greatest width is measured from the lateral aspect of one cerebellar hemisphere to that of the other side (Fig. 2).

The gestational age of the neonates was based on crown rump length measurements determined by the Maternal Fetal Medicine group in early pregnancy. In cases where the crown rump length measurement was not available, the maternal last menstrual period was used to determine the gestational age. The examining radiologist was blinded to the gestational age at the time of the neonatal cerebellar measurement.

The relevant clinical history and details, including the corrected gestational age of the neonates, were obtained from the clinical record. The ultrasounds were performed by either sonologists or sonographers with long-term assignment to neonatal intensive care unit work. All head ultrasounds were reviewed and interpreted on the Picture Archiving and Communication System (PACS) by a single fellowship-trained senior radiologist with 44 years of experience.

A total of 1,260 cases were evaluated. The studies were conducted in different patients. Repeat ultrasound of the same patient was not included in the data. We included neonates less than or equal to (≤) 21 days of life. A subgroup of the exams of those neonates who were ≤10 days of life was evaluated with the entire group and then separately. The reason we used and analyzed both the larger ≤21-day group (*n*=671) and the smaller ≤10-day group (*n*=530) was to see if there was a difference in using the larger group. By noting no significant difference between the two groups, it gives the clinician greater leeway in determining gestational age if the neonate was not sent for an initial head ultrasound until the 11th day of life. This makes the resultant chart more helpful. We excluded neonates greater than 21 days of life to ensure a closer age to the neonate’s birth date and perhaps greater reliability when comparing our measurements to per gestation age weeks found in fetal cerebellar width charts. We excluded neurosonograms/cases with evidence of congenital abnormalities, intraventricular hemorrhage, or other key abnormalities. We excluded any study with technically limited images, particularly those with any asymmetry of the transmastoid view or limited imaging of the cerebellar hemispheres and vermis. A total of 589 cases were excluded. After fulfilling all exclusion criteria, there were 671 cases of neonates ≤21 days of life. Of these 671 cases, 530 cases evaluated were of neonates ≤10 days of life.

We used the fetal chart of transcerebellar measurements related to gestational age developed by Chavez et al. as our gold standard [3].

All statistical analyses were performed using either R or R studio software (version 4.3.3, Boston, MA) [10]. Linear regression was used to compare our data with the existing fetal charts, as well as between our two datasets (neonates ≤21 days and neonates ≤10 days). The transcerebellar measurements were used as the dependent variable while gestational age was used as the independent (predicting) variable.

*Z*-scores were calculated to compare the difference between the two linear regression lines, i.e., whether the regression line is different between our data and the gold standard literature data from Chavez et al. [3] or between data containing neonates ≤21 days versus neonates ≤10 days. Significance levels were set at *P*≤0.05 (two-sided). 95% confidence intervals were calculated by standard methods: the mean±1.96 standard deviations from the mean.

## Results

The transcerebellar measurements were tabulated, depicting the mean values and standard deviations for both the ≤21-day-old and the ≤10-day-old neonatal groups. Table 1 shows the transcerebellar measurements obtained in the ≤21-day-old group. The mean values and standard deviations at each gestational week (between 22 weeks and 40 weeks) as well as the 95% confidence interval have been tabulated. Table 2 shows the transcerebellar measurements in the ≤10-day-old group. The mean values and standard deviations at each gestational week along with the 95% confidence interval have been tabulated.

**Table 1:** Mean values of transcerebellar measurements obtained in neonates on day of life ≤21. The standard deviation and 95% confidence interval at each gestational week are shown. *DOL*, day of life; *GA wks*, gestational age in weeks; *n*, number of patients; *mean*, mean transcerebellar diameter in cm; *SD*, standard deviation; *CI*, confidence interval

DOL ≤21					
GA wks	*n*	Mean (cm)	SD	95% CI lower	95% CI upper
22	15	2.34	0.18	2.24	2.43
23	13	2.53	0.20	2.42	2.65
24	40	2.63	0.21	2.57	2.70
25	26	2.78	0.28	2.67	2.88
26	34	3.00	0.23	2.92	3.08
27	47	3.16	0.30	3.08	3.25
28	63	3.28	0.33	3.20	3.36
29	54	3.49	0.32	3.40	3.57
30	66	3.69	0.36	3.60	3.78
31	38	3.85	0.41	3.72	3.98
32	28	4.10	0.34	3.98	4.23
33	24	4.23	0.42	4.06	4.39
34	34	4.30	0.42	4.15	4.44
35	14	4.55	0.38	4.35	4.75
36	36	4.78	0.34	4.67	4.89
37	41	4.83	0.36	4.72	4.94
38	32	5.01	0.33	4.90	5.12
39	44	5.16	0.39	5.04	5.27
40	22	5.21	0.39	5.04	5.37

**Table 2:** Mean values of transcerebellar measurements obtained in neonates on day of life ≤10. The standard deviation and 95% confidence interval at each gestational week are shown. *DOL*, day of life; *GA wks*, gestational age in weeks; *n*, number of patients; *mean*, mean transcerebellar diameter in cm; *SD*, standard deviation; *CI*, confidence interval

DOL ≤10					
GA wks	*n*	Mean (cm)	SD	95% CI lower	95% CI upper
22	10	2.32	0.21	2.19	2.45
23	9	2.48	0.20	2.35	2.61
24	34	2.61	0.20	2.55	2.68
25	15	2.69	0.29	2.55	2.84
26	24	2.95	0.22	2.86	3.04
27	37	3.09	0.27	3.00	3.18
28	50	3.21	0.30	3.13	3.29
29	39	3.42	0.30	3.32	3.51
30	55	3.63	0.33	3.54	3.72
31	28	3.78	0.39	3.64	3.93
32	20	3.97	0.27	3.86	4.09
33	14	4.12	0.45	3.88	4.35
34	27	4.26	0.41	4.11	4.42
35	10	4.62	0.40	4.37	4.86
36	29	4.76	0.37	4.63	4.90
37	36	4.81	0.34	4.70	4.92
38	29	5.02	0.33	4.90	5.14
39	43	5.16	0.39	5.04	5.27
40	21	5.20	0.40	5.03	5.37

**Fig. 3:**
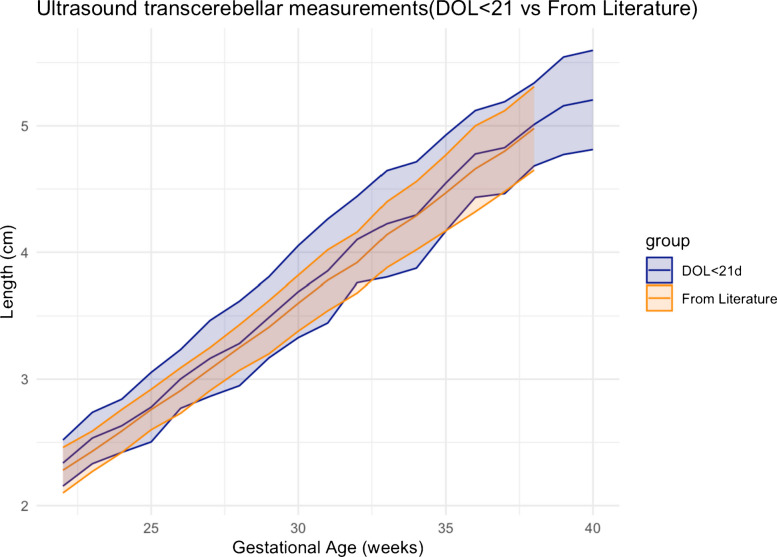
Linear regression plot comparing transcerebellar diameter in day of life ≤21 and literature data. The data in literature includes neonates only up to 38 weeks of gestation, whereas our data includes neonates up to 40 weeks of gestation. There is a 0.15 *P*-value suggesting no significant difference between gestational age determination by transcerebellar diameter for neonates ≤21 days and the gestational age determination by the gold standard fetal transcerebellar diameter chart of Chavez et al. [3]. *DOL*, day of life

**Fig. 4:**
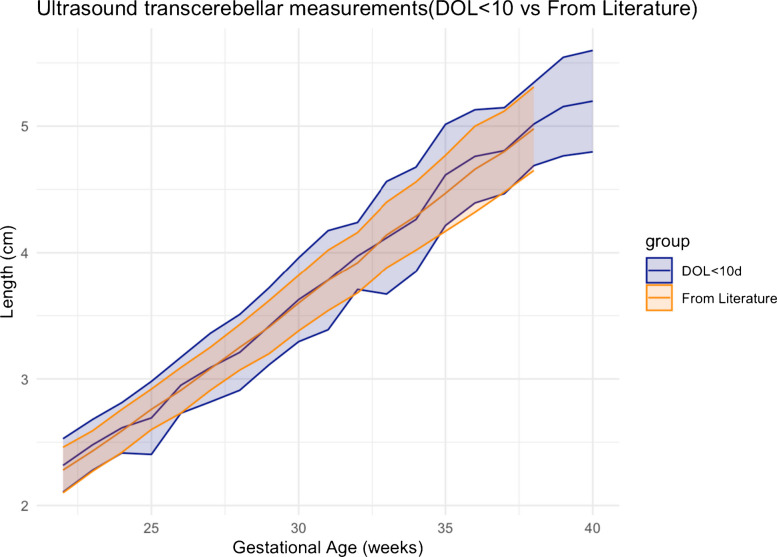
Linear regression plot comparing transcerebellar diameter in day of life ≤10 and literature data. The data in literature includes neonates only up to 38 weeks of gestation, whereas our data includes neonates up to 40 weeks of gestation. There is a 0.87 *P*-value suggesting no significant difference between gestational age determination by transcerebellar diameter for neonates ≤10 days and the gestational age determination by the gold standard fetal transcerebellar diameter chart of Chavez et al. [3]. *DOL*, day of life

Linear regression statistical methodology used to compare whether the transcerebellar diameter results of day of life ≤10 and transcerebellar diameter results for those of day of life ≤21 are different than the transcerebellar diameters reported in the literature [3] is plotted with the 95% confidence interval plotted along with the mean values. By *Z*-score comparison, there was no significant difference between the measurements of day of life ≤21 compared to the measurements reported in the literature (*P*=0.15) (Fig. 3). There was no significant difference between measurements of day of life ≤10 with the fetal measurements reported in the literature (*P*=0.87) (Fig. 4). The gold standard literature data [3] has measurements only up to 38 weeks of gestation age, whereas our data is up to 40 weeks of gestation age. As a result, the graphs in Figs. 3 and 4 demonstrate only our data at 39 weeks and 40 weeks of gestation, without overlapping from literature data.

**Fig. 5:**
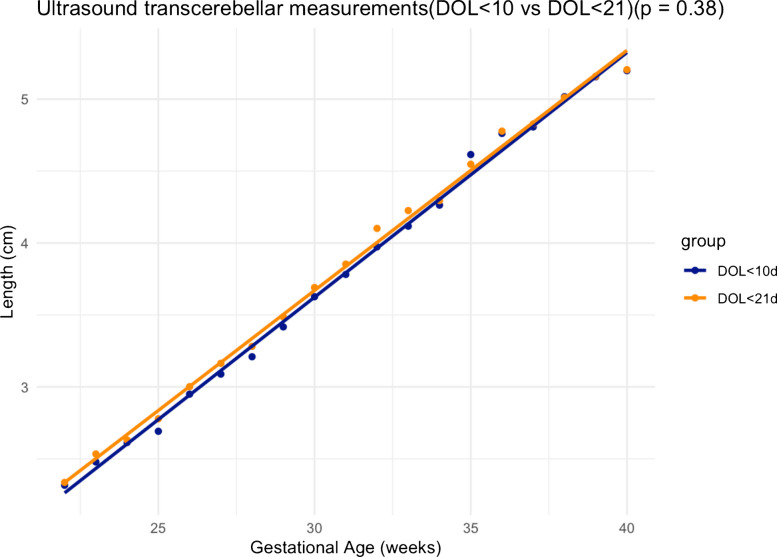
Linear regression plot comparing transcerebellar diameter in day of life ≤21 and day of life ≤10. A *P*-value of 0.38 indicates no significant difference between gestational age determination by transcerebellar measurements in the study whether the neonatal measurement was determined in the ≤10-day-old group versus ≤21-day-old group. *DOL*, day of life

The same *Z*-score method used to compare the two sets of measurements based on linear regression demonstrated no significant difference (*P*=0.38) in transcerebellar diameter measurements obtained for the day of life ≤10 and the day of life ≤21 groups (Fig. 5).

## Discussion

Our work shows that transcerebellar measurements obtained by neonatal transmastoid fontanelle sonography can help determine the gestational age. Knowing the accurate gestational age can assist the clinicians in appropriate treatment and timely interventions of neonates.

Currently, the New Ballard Score [6–8] is the most widely accepted method for gestational age determination in neonates. It is a clinical method based on physical and neuromuscular maturity and can be reliably used up to 4 days after birth, most accurate within 48 h. Physical maturity is assessed by evaluation of the skin, lanugo, plantar surface, breast, eye/ear, and genitals. The neuromuscular maturity is assessed by posture and multiple clinical examinations like arm recoil and heel to toe. The New Ballard Score is a subjective interpretation of the clinical examination and has reduced reliability, especially in very preterm neonates [9]. It can under- or overestimate the gestational age. The score depends on the neuromuscular function of the neonate, which can be altered due to exposure to maternal medications. Extreme prematurity also limits the physical examination. Singhal et al. noted that the New Ballard Score overestimates the gestational age in very small for gestational age neonates [11].

The transcerebellar diameter measurement of a fetus by antenatal obstetric ultrasound is an accepted parameter for determining the gestational age [3–5]. There are published articles depicting transcerebellar diameter nomograms through different gestational ages [3, 5]. The largest study performed to date has been published by Chavez et al. in 2003 [3]. This was a retrospective study, which included approximately 24,000 cases, and provided normative data of fetal cerebellar growth throughout gestation.

The use of neurosonography through an anterior fontanelle approach using a static B mode scanner was first described by two separate groups, Babcock DS et al. and Ben-Ora et al. in the late 1970s and early 1980 s [12, 13]. Real-time B mode imaging of the neonatal brain via an anterior fontanelle approach was first described by Grant et al. in 1981 [14]. Besides the use of the anterior fontanelle, additional imaging of the brain has been reliably performed via any open suture [15]. The use of the mastoid fontanelle as an additional acoustic window was first presented by Mata et al. at the May 1996 International Pediatric Radiology meeting. Buckley et al. published a pictorial article in AJR in 1997 demonstrating the usefulness of mastoid views for evaluating the posterior fossa and midbrain anatomy in premature and term neonates [16]. Although developed to evaluate for intracerebellar hemorrhage in neonates, the mastoid view provides excellent evaluation of the anatomy of posterior fossa structures [17].

In 2000, Davies et al. [18] published a mixed retrospective and prospective study of Australian neonates between 23 weeks and 32 weeks of age, with a goal of noting whether transcerebellar diameter can be used for gestational age determination. They found a close relationship between transcerebellar diameter and gestational age, with transcerebellar diameter increasing linearly with gestational age. They created a percentile chart. This study is limited compared to our study and included a total of 221 neonates only up to 32 weeks of gestation and in an Australian population only. Our study is the first North American study of a larger population and a broader range of weeks of gestation, and includes the creation of a table for gestational age determination by transcerebellar diameter. It includes neonates between 22 weeks and 40 weeks of gestation. The total subjects included in our study are 671, approximately three times larger than the prior Australian study.

In the past, there was a proof-of-concept paper by Aziz et al. [19] to determine if the transcerebellar measurements obtained by transmastoid ultrasound could be compared to crown rump length in gestational age estimation. This study concluded that postconceptual gestational age estimation by neonatal transmastoid transcerebellar diameter was highly correlated with that of first-trimester ultrasound. Only 62 cases were included in this study. Our study has a larger number of cases, and an attempt is to create a usable chart for neonatal gestational age determination by transcerebellar diameter measurement.

Our study is an improvement over Davies et al. [18] in that it consists of a retrospective analysis of a larger group of patients, 671 head ultrasounds of patients ≤21 days of life, 530 of which were below day of life 10. We arbitrarily chose 21 days beyond birth as a cutoff for case inclusion in case there could be significant postnatal changes in neonatal cerebellar growth that would make our measurements unreliable. We found no difference between measurements for day of life ≤10 and those for day of life ≤21. Based on this, our work and generated chart include determined transcerebellar measurements among neonates evaluated at 21 days of life or younger. These were confirmed as reliable when compared to antenatally obtained measurements in fetuses of similar ages using the fetal transcerebellar diameter gold standard charts. Our work could provide a reliable estimation of gestational age in neonates. In short, our chart of transcerebellar diameter measurements for day of life ≤21 (Table 1) can help with gestational age determination.

As with all studies, potential weaknesses of this study exist. This includes the inability to evaluate neonates less than 22 weeks of gestation because of limited numbers due to reduced likelihood of viability. We determined gestational age for weeks of life within our neonatal intensive care unit studied group, but days of life rather than weeks of life might be a more exacting methodology for future research.

There is a limitation to the use of transcerebellar measurement when evaluating through a lateral approach, which is what occurs when using the mastoid fontanelle from either the right side or left side. We limited this weakness by not including any cerebellar imaging that was asymmetric, probably related to the technique or obstructing hardware.

Our study establishes that the transcerebellar measurements of neonates between 22 weeks and 40 weeks obtained by transmastoid neurosonography are statistically as reliable as the accepted measurements in fetuses.

## Conclusion

Gestational age determined in neonates by transmastoid measurement of their cerebellar diameters in the first 3 weeks of life is similar to those of fetuses of equivalent gestational ages using the standard fetal chart of Chavez et al. [3].

We have created a table (Table 1) to determine gestational ages in neonates based on neonatal transcerebellar diameters. This will hopefully aid gestational age determination in neonates born of unknown gestational age.

## Data Availability

No datasets were generated or analysed during the current study.
